# The effectiveness and safety of direct-acting antivirals for hepatitis C virus treatment: A single-center experience in Saudi Arabia

**DOI:** 10.1016/j.jsps.2022.07.005

**Published:** 2022-07-25

**Authors:** Sumaiah J. Alarfaj, Abdullah Alzahrani, Anfal Alotaibi, Malak Almutairi, Mashael Hakami, Njood Alhomaid, Noori Alharthi, Ghazwa B. Korayem, Abdullah Alghamdi

**Affiliations:** a Department of Pharmacy Practice, College of Pharmacy, Princess Nourah bint Abdulrahman University, Riyadh, Saudi Arabia; b Department of Pharmacy, Security Forces Hospital, Riyadh, Saudi Arabia; c Internal Medicine Department, Security Forces Hospital, Riyadh, Saudi Arabia

**Keywords:** Hepatitis C virus, Direct-acting antivirals, Saudi population, Sustained virologic response, Genotype 4, Effectiveness, DAA, direct-acting antiviral agent, DSV, dasabuvir, LDV, ledipasvir, SOF, sofosbuvir, OBV, ombitasvir, PTV, paritaprevir, r, ritonavir, RBV, ribavirin, GT, genotype, HCV, hepatitis C virus

## Abstract

**Background:**

The introduction of direct-acting antivirals (DAA) to treat the hepatitis C virus (HCV) overcame many drawbacks of interferon-based therapy. DAA achieved sustained viral response (SVR) rates above 90% and overcame many drawbacks of pegylated interferon regimens.The HCV genotype (GT) distribution varies by geographical area, with GT-4 being most prevalent in the Middle East region, including Saudi Arabia. Yet, the real-world evidence about using DAAs in the Saudi population is limited.Thus, the aim of this study to investigate the effectiveness and safety of DAAs in Saudi patients with HCV infection.

**Methods:**

A retrospective cohort study included patients treated with DAAs from 2015 to 2017 at a tertiary care hospital in Riyadh, Saudi Arabia. All patients with HCV treated with either ledipasvir plus sofosbuvir (LDS/SOF) ± ribavarin (RBV) or ombitasvir-paritaprevir-ritonavir (OBV/PTV/r) ± dasabuvir (DSV) ± RBV were included. Using a per-protocol analysis, the effectiveness outcome was the end-of-treatment response (EOTr) and Sustained virologic reponce12 weeks after competing the regimen (SVR12). The secondary safety outcome was the adverse event related to the therapy reported by the patients.

**Results:**

A total of 97 patients were included; with the majority infected with GT-4 (64 %), followed by GT-1 (18 %), in addition to 8 % having a mixed GT (1 + 4). The EOTr and SVR12 rates were 98 % and 96 %, respectively. SVR12 was 94.4 % within the LDS/SOF ± RBV group and 97.7 % within the OBV/PTV/r ± DSV ± RBV group. Only 4 % had a response failure due to relapse or breakthrough, and all were infected with mixed GT1 + 4. Medications were well tolerated with minimal side effects, including vomiting, nausea, and weakness.

**Conclusion:**

DAAs regimens are associated with high rates of SVR12 and are well tolerated with a good safety profile in Saudi HCV-infected patients.

## 1 Introduction

Hepatitis C virus (HCV) infection is a major cause of liver cirrhosis and is the most common indication for liver transplantation worldwide (Axley et al., 2018, Bhamidimarri et al., 2017).

Globally, 177.5 million people are infected with HCV, including several genotypes (GTs) that vary across different geographic areas. HCV genotype (GT) 1 is the most prevalent worldwide (49.1 %), followed by GT-3 (17.9 %), 4 (16.8 %) and 2 (11.0 %). GT 5 and 6 are responsible for the remaining < 5 % (Petruzziello et al., 2016). However, the highest prevalence of HCV infection is presented in the Eastern Mediterranean countries, with around 2.3 % for 15.2 million cases(Centre for Disease Analysis, 2017). The prevalence of anti-HCV antibodies in the Kingdom of Saudi Arabia (KSA) is estimated to be 0.7 %, and approximately 70 % of these individuals have an active infection (Aljumah et al., 2016).

The most common GT infection in Saudi Arabia is GT-4 at 65 %, followed by GT-1 at 23 %. An apparent decline in the rate of HCV GT-4 has been demonstrated among the younger age group in KSA, in which they have an increased rate toward the global trend of HCV GT-1 rather than GT-4. However, HCV GT-4 still shows an overall high prevalence in KSA (Bawazir et al., 2017).

Until a few years ago, the only treatment strategy was a combination of pegylated interferon and ribavirin for 24 or 48 weeks, depending on the genotype. This regimen showed a modest response rate of up to approximately 55 % and was associated with low tolerability due to a serious side effect profile (González-Grande et al., 2016). In the last few years, the standard of care for HCV infection has evolved substantially. It currently includes the introduction of first-generation direct-acting antivirals (DAAs). In 2011, boceprevir and telaprevir were the first DAAs agents approved for the treatment of GT-1, in combination with pegylated interferon and ribavirin (Almeida et al., 2021).The introduction of DAA increased the sustained viral response (SVR) rates above 95 %, offered excellent tolerability, shorter duration of treatment, less toxicity, and regimens free of pegylated interferon (Almeida et al., 2021, Bansal et al., 2015). These DAAs include nucleotide nonstructural (NS) 3/4A protease inhibitors, NS5B polymerase inhibitors, and NS5A polymerase inhibitors. Most of the DAAs are used to treat more than one HCV GT, and all have proven effective and well-tolerated in clinical studies (Bansal and Morris, 2019).

The Epideiology of HCV genotypes differ throughout the world, with (65.3 %) of worlds GT-4 being in North African and middle east region, followed by (28.1 %) in Africa (Petruzziello et al., 2016). Yet, only few efficacy studies are done in this area of the world, and non include Saudi patients. (Di Biagio et al., 2018) The World Health Organization (WHO) aims to eliminate HCV worldwide by 2030, and this was adopted and reflected in the Saudi Arabian Ministry of Health’s goals (Altraif, 2018, Waheed et al., 2018). However, real-world evidence about the safety and effectivness of DAAs in the Saudi population is limited. Therefore, this study aims to investigate the effectiveness and safety of DAAs in Saudi patients with HCV infection.

## 2 Method

### 2.1 Study design and setting:

This is a retrospective cohort study assessing patients treated with DAA for treatment of chronic HCV in a tertiary care hospital in central Saudi Arabia, including the data of patients treated for HCV between April 2015 and June 2017. The data were collected from the patient’s health records of Security Forces Hospital, wich is a tertiary care military hospital in Riyadh, Saudi Arabia. It includes 532 beds that serve military personnel and their families from all over Saudi Arabia.

### 2.2 Study participants

We included all adult patients aged 18 years or above diagnosed with chronic HCV infection, regardless of the treatment history (naïve, or experienced with interferon-based therapy) or liver fibrosis stage. Patients who had started HCV treatment but did not have any information about the outcome (missing data or lost to follow-up) were excluded from the analysis.

The decision of the treatment regimen was left to the treating physicians following the AASLD-IDSA guidelines for Hepatitis C virus (Chung et al., 2015) then the Saudi Association for the Study of Liver Disease (SASLT) guideline (Alghamdi et al., 2016).

Guidelines view the recommended treatment regimens for each genotype as equivalent and the decition of which to use involeve Drug interactions, and availability of regimen.

In this cohort study, 2 core DAA regimes were used in the hospital to treate HCV patients, bothe guidline recommend their use similarly. Patients were treated with either ledipasvir plus sofosbuvir (LDS/SOF) ± ribavarin (RBV) or ombitasvir-paritaprevir-ritonavir (OBV/PTV/r) ± dasabuvir (DSV) ± RBV depending on GT, treatment history, and cirrhosis.

### 2.3 Data collection and outcomes

All patients who were treated with DAA for HCV infection during the study period were included. Patient information and outcomes were collected at baseline from the patient’s electronic medical records through Research Electronic Data Capture (REDCap®) 7.3.6 software by a few investigtirs. Another investigator randomly checked a sample of 10 % of the data to assure no errors were made in data collection The collected information included the patient’s baseline characteristics, such as age, gender, comorbidities, HCV GT, HCV Ribonucleic acid (RNA) viral loud, treatment history (naïve, or experienced with interferon-based therapy), and baseline liver assesments including Child-pugh score and fibrosis level. In addition, we collected data about the HCV treatment regimen, treatment duration, and otcomes.

#### 2.3.1 Outcomes

The effectiveness of the DAA regimens was determined using the level of HCV RNA using the polymerase chain reaction (PCR) test, which was checked immediately after finishing the treatment course, to evaluate the end-of-treatment response (EOTr), and 12 weeks after completing the treatment regimen, to evaluate the Sustained virologic response (SVR12).

Patients who had an increase in HCV RNA to 100 IU/mL or higher at any time during treatment were classified as having a “breakthrough infection.” Patients were categorized as having HCV “relapse” if they completed taking the study’s regimen (mentioned below) with undetectable HCV RNA during the regimen treatment period, then HCV RNA was detected 12 weeks after completing the regimen.

The primary effectiveness outcome was the proportion of patients achieving SVR12, otherwise known as cure rate. While, the secondary effectiveness outcomes was the proportion of patients achieving EOTr rate.

The safety outcome was the adverse event reported by the patients from the beginning of treatment up to 30 days following the final dose of the study regimen.

### 2.4 Statistical analysis

We performed a per-protocol data analysis. Descriptive continuous data were reported as mean ± standard deviation and compared using student *t*-test for normally distributed variables, and Mann Whitney *U* test for non-normal distrubution. Variable distribution normality was verified using the Shapiro-Wilk test. Categorical variables were presented as frequency and percentage, and compared using Chi-square or Fisher’s exact test as appropriate. All reported statistical tests were two-sided, and p < 0.05 was set as statistically significant. Data were analyzed using SPSS version 24 (SPSS Inc., Chicago, IL, USA).

The study was reviewed and approved by the Institutional Review Board of Security Forces Hospital in Riyadh, Saudi Arabia. (IRB no: H-01-R069).

## 3 Results

### 3.1 Patient characteristics

A total of 104 patients met the inclusion criteria. However, seven patients were excluded from the analysis of primary and secondary outcomes due to lost follow-up after the end of treatment and insufficient documentation of SVR12. Thus, a total of 97 patients with HCV infection with or without liver cirrhosis were included in the per-protocol analysis. The mean age of the patients was 52.7 ± 14.9 years, out of which 54.8 % were female, and 50 % were treatment-experienced with the interferon-based regimen.

The number of patients treated with LDS/SOF were comparable to those treated with OBV/PTV/r at 54 and 43, respectively. The baseline characteristics including age, gender, comorbidities, fibrosis, and treatment history of patients receiving the two treatment regimens, LDS/SOF and OBV/PTV/r, were comparable.

The most observed genotype in all study subjects was GT-4 at 63.7 %, followed by GT-1 at 24 %. Further detailes about baseline charactaristics are presented in Table 1.

**Table 1:** Baseline characteristics of included patients treated for HCV (n = 97).

	Total (n = 97)	Ledipasvir + Sofosbuvir (n = 54)	Ombetasvir + Paritaprevir + Ritonavir (n = 43)	P-value
Age (years), mean ± SD	52.5 ± 15.1	54.1 ± 14.8	50.5 ± 15.4	0.246
Gender, n, (%)				
Female	52 (53.60)	28 (51.90)	24 (55.80)	0.838
Male	47 (46.40)	26 (48.10)	19 (44.20)	
Genotype, n, (%)				
Genotype 1a	16 (16.5)	9 (16.)	7 (16.3)	0.240
Genotype 1b	8 (8.2)	4, (7.4)	4 (9.3)	
Genotype 3	1 (1.00)	1 (1.9)	0 (0)	
Genotype 4	62 (63.90)	31 (57.4)	31 (72.1)	
Mixed 1 + 4	8 (8.20)	7 (13)	1 (2.3)	
Unknown	2 (2.10)	2 (3.7)	0 (0)	
RNA level, viral load IU/ml	21.4. × 10^5^ ± 29.6 × 10^5^	18.2 × 10 ^5^ ± 22.7 × 10 ^5^	25.4 × 10 ^5^ ± 36.3 × 10 ^5^	0.233
>= 800,000 IU/ml, n, (%)	52, (53.6)	28, (51.9)	24 (55.8)	0.838
BMI, mean ± SD	30.5 ± 7.2	30.2 ± 6.5	30.0.8 ± 8.1	0.730
Comorbidities, n, (%)				
DM	40 (41.2)	24, (44.4)	16 (37.2)	0.536
HTN	37 (38.1)	20 (37)	17 (39.5)	0.836
CAD	10 (10.3)	7 (13)	3 (7)	0.505
CKD	7 (7.2)	2 (3.7)	5 (11.6)	0.236
HBV	1 (1)	0 (0)	1 (2.3)	0.443
HIV	0 (0)	0 (0)	0 (0)	
Transplant	7 (7.2)	5 (9.3)	2 (4.7)	0.458
Other	41 (42.3)	27 (50)	14 (32.6)	0.100
None	27 (27.8)	14 (25.9)	13 (30.2)	0.655
Fibrosis, n, (%)				
F 0–3	62 (63.9)	34 (63)	28 (65.1)	0.835
F 4	35 (36.1)	20 (37)	15 (34.9)	
Child-Pugh, n, (%)				
A	28 (80)	17 (85)	11 (73.3)	0.672
B	7 (20)	3 (15)	4 (26.7)	
Treatment Hx, n, (%)				
Experienced	49 (50.5)	24 (44.4)	25 (58.1)	0.222
Naïve	48 (49.50)	30 (55.6)	18 (41.9)	
Antivirals added to the core Regimen, n, (%)				
Dasabuvir	10 (10.3)	0, (0)	3 (6.97)	0.003
Ribavirin	55 (56.7)	16 (29.60)	32 (74.41)	<0.001
Dasabuvir and Ribavirin	7 (7.2)	0 (0)	7 (16.28)	0.003

**Hx:** history, **BMI:** body mass index, **HIV:** human immunodeficiency virus, **HBV:** hepatitis B virus, **CKD:** chronic kidney disease, **CAD:** coronary artery disease, **HTN:** hypertension, **DM**: diabetes mellitus.

### 3.2 Outcomes:

#### 3.2.1 Effectiviness

Overall, the EOTr rate and cure rate were high at 96.9 % and 95.9 %, respectively. The cure rate in the LDS/SOF group was 94.4 % compared to 97.7 % in the OBV/PTV/r group (*P* = 0.627), as presented in Table 2. All patients with HCV genotypes 1a, 1b, 3, and 4 achieved SVR12, while patients with mixed GT1 + 4 achieved SVR12 rates of 55 % (5/8)**.** Breakthrough and relapse rates were both low, at 2 % for each group.

**Table 2:** Comparison of treatment responce with Ledipasvir + Sofosbuvir vs Ombetasvir + Paritaprevir + Ritonavir.

	Total (n = 97)	Ledipasvir + Sofosbuvir (n = 54)	Ombetasvir + Paritaprevir + Ritonavir (n = 43)	P-value
Duration, n, (%)				
8 weeks	1 (1)	1 (1.9)	0 (0)	0.064
12 weeks	91 (93.8)	48 (88.9)	43 (100)	
24 weeks	5 (5.2)	5 (9.3)	0 (0)	
EOTr, n, %				
Achieved	94 (96.9)	52 (96.3)	42 (97.7)	0.333
Not achieved	2 (2.1)	2 (3.7)	0 (0)	
SVR_12, n, %				
Achieved	93 (95.9)	51 (94.4)	42 (97.7)	0.627
Not achieved	4 (4.1)	3 (5.6)	1 (2.3)	

**Hx:** history, **BMI:** body mass index, **HIV:** human immunodeficiency virus, **HBV:** hepatitis B virus, **CKD:** chronic kidney disease, **CAD:** coronary artery disease, **HTN:** hypertension, **DM**: diabetes mellitus, **EOTr:** end of treatment response, **SVR_12:** sustained virological response after 12 weeks of completing the regimen.

In this treatment cohort of 97 patients, 64 % of patients received RBV combined with the DAA treatment.

#### 3.2.2 Subcohort analysis of patients receiving ledipasvir + sofosbuvir

**Fig. 1:**
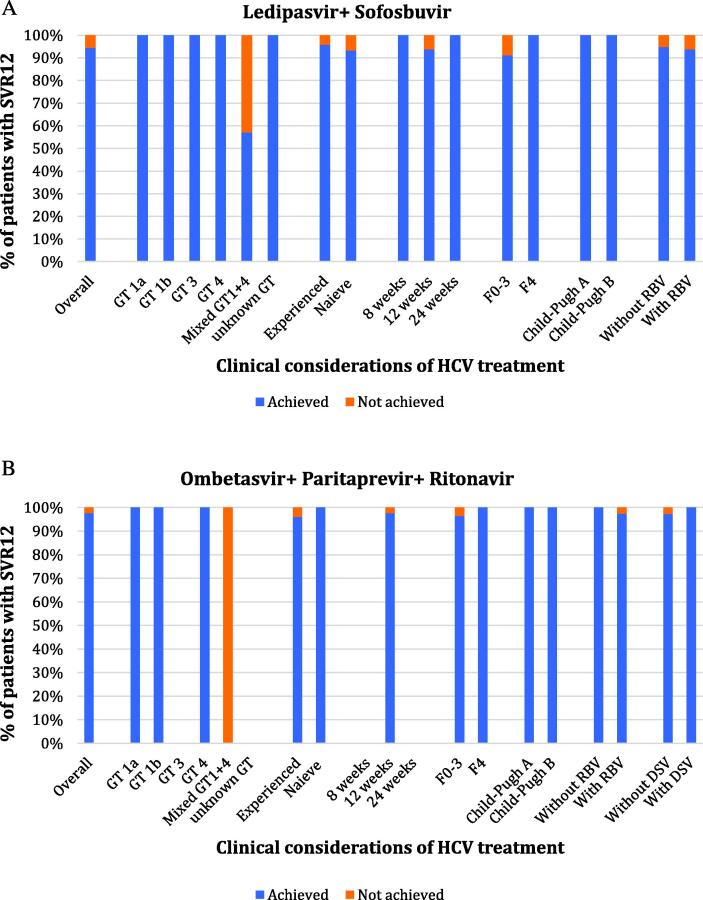
Sustained virologic response rate 12 weeks after completing the treatment regimen (SVR12) for treatment with (A) Ledipasvir + Sofosbuvir; and (B) Ombetasvir + Paritaprevir + Ritonavir, the effect of clinical considerations of HCV on response rate.

Overall, EOTr and SVR12 rates were high at 96.3 % (52/54) and 94.4 % (51/54), respectively. Only one patient was treated with an eight-week regimen, and five patients were treated with a 24-week regimen,further details are depicted in Table 1, Table 2. Overall, 45 % (3/7) of patients with mixed GT1 + 4 did not achieve SVR. Fig. 1.A describes SVR for treatment with ledipasvir + sofosbuvir and the effect of clinical characteristics.

#### 3.2.3 Subcohort analysis of patients receiving ombetasvir + paritaprevir + ritonavir

Overall, EOTr and SVR12 rates were high at 97.7 % (42/43) and 97.7 % (42/43), respectively. All patients within this subcohort received a 12-week regimen. DSV ± ribavarin was added to the regimen in 23.3 % of patients. Ribavarin alone was added to the regimen in 74.4 % of the patients.,further details are depicted in Table 1, Table 2. Fig. 1.B describes the SVR for treatment with ombetasvir + paritaprevir + ritonavir and the effect of clinical characteristics.

#### 3.2.4 Subcohort analysis of GT4

All patients with this genotype were treated successfully (SVR12 100 %) with either LDS/SOF ± RBV (50 %) or OBV/PTV/r ± RBV (50 %). Patients with GT4 required fewer kidney transplantation (P 〈0 0 7), however, this did not affect SVR12 as detailed in Table 3.

**Table 3:** Comparison of baseline characteristics of patients with genotype 4 compared to other genotypes (1, 3).

	Total (N = 87*)	Other genotypes (n = 25)	Genotype 4 (n = 62)	P value
Age(years), mean ± SD	51.7 ± 15.2	50.1 ± 13.9	52.4 ± 15.7	0.528
Gender n, (%)				
Female	48 (55.2)	15 (60	33 (53.2)	0.638
Male	39 (44.8	10 (40)	29 (46.8)	
Viral load, IU/ml, n (%)	18.0 × 10 ^5^ ± 29.9 × 10 ^5^	22.0 × 10 ^5^ ± 37.4 × 10 ^5^	19.6 × 10 ^5^ ± 26.7 × 10 ^5^	0.736
≥ 800,000	44 (50.6)	13 (5)	31 (50)	1
< 800,000	43 (49.4)	12 (48)	31 (50)	
BMI n, (%)	30.7 ± 7.3	28.4 ± 6.0	31.8 ± 7.7	0.059
Underweight	1 (1.3)	0, (0	1 (1.9)	0.110
Normal	19 (25)	10, (41.7)	9 (17.3)	
Overweight	17 (22.4)	5 (20.8)	12 (23.1)	
Obese	39 (51.3)	9 (37.5)	30 (57.7)	
Comorbidities n, (%)				
DM	35 (40.2)	12 (48)	23 (37)	0.469
HTN	34 (39.1)	11 (44)	23 (37.1)	0.630
CAD	9 (10.3)	3 (12)	6 (9.7)	1
CKD	7 (8)	4 (16)	3 (4.8)	0.185
HBV	1 (1.1)	1 (4)	0 (0)	0.287
HIV	0 (0)	0 (0)	0 (0)	
Transplant	6 (6.9)	5 (20)	1 (1.6)	0.007
Other	37 (42.5)	13 (52)	24 (38.7)	0.339
None	24 (27.6)	5 (20)	19 (30.6)	0.429
Fibrosis n, (%)				
F0-3	54 (62.1)	15 (60)	39 (62.9)	0.812
F4	33 (37.9)	10 (40)	23 (37.1)	
Child-Pugh n, (%)				
A	54 (62.1)	15 (60)	39 (62.9)	1
B	33 (37.9)	10 (40)	23 (37.1)	
Treatment history n, (%)				
Experienced	44 (50.6)	13 (52)	31 (50)	1
Naïve	43 (49.4)	12 (48)	31 (50)	
Core Regimen n, (%)				
Ombetasvir + Paritaprevir + Ritonavir	42(48)	11 (44)	31 (50)	0.643
Ledipasvir + Sofosbuvir	45 (51.7)	14 (56)	31 (50)	0.643

*N = total included patients minus mixed and unknown genotypes. **Hx:** history, **BMI:** body mass index, **HIV:** human immunodeficiency virus, **HBV:** hepatitis B virus, **CKD:** chronic kidney disease, **CAD:** coronary artery disease, **HTN:** hypertension, **DM**: diabetes mellitus.

#### 3.2.5 Safety

Overall, treatments were tolerated very well. Only 2.9 % of all included patients reported having minor side effects, including nausea, vomiting, and weakness.

## 4 Discussion

To our knowledge this is the first study to report outcomes of HCV treatment with DAA in government funded hospitals. Findings of this study are favorable, with a SVR12 of 95.9 % of all patients who completed the treatment regimen. Within the GT-4 subgroup, SVR12 was 100 %. These high cure rates are consistant with real-world data reported in medile east contries (Ahmed et al., 2018, El Kassas et al., 2022). In this cohort, all patients who did not achieve SVR12 were mixed GT1 + 4 (4/97). The current study highlite the high HCV cure rate achived with DAA in Saudi population and identify mixed GT infection as having a higher chance of treatment failer in this cohort of patients. Our results are similar to a previous retrospective cohort study conducted using health insurance data from the private health sector in KSA (Hashim et al., 2020). In that study, patients treated with DAA-based regimens showed an overall SVR rate of 97 %, and predictors of SVR failure included Egyptian patients of mixed GT1 + 4 (Hashim et al., 2020). Data regarding patients with mixed GTs are sparse due to its rare occurrence, and future studies should investigate if those patients may benefit from the recently FDA approved pangenotypic regimens of DAA(AASLD/IDSA, 2021).

Funding of this study indicate that SVR12 was comparable between OBV/PTV/r ± RBV ± DSV and LDS/SOF ± RBV, at 97.7 % and 94.4 % respectively. These results are comparable to an observational study conducted in Turkey compared LDS/SOF and OBV/PTV/r + RBV ± DSV combination therapy in more than 4,000 patients who reported SVR of 99 % vs 97.5 %, respectively (Degertekin et al., 2020).

Side effects reported in our study were minimal, including nausea, vomiting, and weakness. These findings in the Saudi population are consistent with global findings (McGlynn et al., 2019, Scavone et al., 2016).

This study is the first study to investigate the effectiveness and safty of DAA in Saudi population exhbiting the efforts of the Saudi government eliminate HCV. The results of this study as adds to the evidence that supports DAAs’ effectiveness and safety particularly in the Saudi population. However, it still has some limitations that may limit the generalizability of the results. First, it is a single-centered retrospective cohort study, and is subjected to known bias in this design including slection bias, and un-measurable confounders. Second, the study sample size is relatively small yet comparable to previous studies (Chen et al., 2020, Degasperi et al., 2019, Loo et al., 2019). Therefore, there is a need for a larger and multicenter study in KSA to confirm DAA effectiveness for HCV treatment.

Currently, with the introduction of newer generation pan-genotypic oral DAA, SVR12 rates are higher and predictive factors for SVR12 success might vary. Therefore, larger real-world obesrvational studies assessing their effectiveness and predictors of treatment success remain needed.

## 5 Conclusion

Findings of this study indicate that DAAs regimens are associated with high SVR12 rates of upto 96 % and good safety profile in Saudi population. Nonetheless, patients with mixed GT1 + 4 were associated with higher chances of treatment failure. These findings can support the decision of HCV treatment in KSA. However, larger studies in KSA are needed to further investigate HCV treatment outcomes, identify predictors of response failure, and determine the best approach in mixed GT1 + 4 patients.

## Disclaimer

The contents of this manuscript are solely the authors’ views and may not be understood or quoted as being made on behalf of or reflecting the position of the Saudi Food and Drug Authority.

## Declaration of Competing Interest

The authors declare that they have no known competing financial interests or personal relationships that could have appeared to influence the work reported in this paper.
